# Topological data analysis of the firings of a network of stochastic spiking neurons

**DOI:** 10.3389/fncir.2023.1308629

**Published:** 2024-01-04

**Authors:** Xiaotian Bai, Chaojun Yu, Jian Zhai

**Affiliations:** School of Mathematical Sciences, Zhejiang University, Hangzhou, China

**Keywords:** topological data analysis, persistent homology, spiking neural network, Betti curves, criticality

## Abstract

Topological data analysis is becoming more and more popular in recent years. It has found various applications in many different fields, for its convenience in analyzing and understanding the structure and dynamic of complex systems. We used topological data analysis to analyze the firings of a network of stochastic spiking neurons, which can be in a sub-critical, critical, or super-critical state depending on the value of the control parameter. We calculated several topological features regarding Betti curves and then analyzed the behaviors of these features, using them as inputs for machine learning to discriminate the three states of the network.

## 1 Introduction

The critical brain hypothesis that the brain operates near a critical phase transition point for optimal information processing functions (Beggs and Plenz, 2003; Kinouchi and Copelli, 2006; Beggs, 2008; Shew et al., 2011; Shew and Plenz, 2013) has been studied for 20 years. Though there are controversies (Touboul and Destexhe, 2010, 2017; Destexhe and Touboul, 2021), this hypothesis is still well supported (Fontenele et al., 2019; Plenz et al., 2021; Beggs, 2022). Therefore, it remains an important problem about how to determine whether a neural system is critical or not. Usually, it is assessed via neural avalanches (Beggs and Plenz, 2003). When the distributions of avalanche size and duration follow power-law and the corresponding exponents obey the crackling scaling relation (Muñoz et al., 1999; Sethna et al., 2001), the neural system is considered critical. However, as far as we know, there are yet no sufficient conditions for the criticality of neural systems, and much more studies should be devoted to finding more methods for assessing the criticality in neuroscience.

On the other hand, recently applied topology has become an important tool in neuroscience and found numerous applications in several aspects, for its convenience in analyzing and understanding the structure and dynamic of neural systems. By constructing simplicial complex from brain activity data, one can identify the topological features of functional modules, hierarchical organization, and diseases in brain. For example, in 2015, the Blue Brain Project reconstructed a data-driven model of the rat neocortical microcircuitry which supports the diverse information processing (Markram et al., 2015), and later in 2017, they did a further topological analysis on the microcircuitry and found that the high-dimensional topological structure appeared and disintegrated with the spatial-temporal stimulus (Reimann et al., 2017). Moreover, by applying the persistent homology and nerve theorem, one can extract global features of the external environment by analyzing the co-firing activities of cells (Curto and Itskov, 2008; Giusti et al., 2015). For instance, in the hippocampus, by modeling the place cells with different firing parameters (i.e., firing rates, place field size, and number of neurons), the computation of persistent homology on the co-firing activities gives a topological information about the environment and the learning region wherein the corresponding parameters can make the model generate stable topological representation (Dabaghian et al., 2012). Furthermore, many other topological analysis methods and biophysiological factors were considered in the spatial learning in the hippocampus. Arai et al. (2014) explored the effects of θ phase precession on spatial learning, and later on, they evaluated the effect of decaying connections between hippocampus neurons (Babichev et al., 2018) and the replay mechanism (Babichev et al., 2019) using a novel topological technique called zigzag persistent homology. Topological data analysis (TDA) is also successfully applied in other spatial cognitive systems such as head-direction cells, grid cells, and conjunctive cells that span low-dimension topological structures embedded in high-dimensional neural activity space (Curto, 2016; Kang et al., 2021; Gardner et al., 2022) in which persistent cohomology techniques are used. Moreover, persistent homology has been used to analyze the spiking data generated from artificial neural network (Spreemann et al., 2018; Bardin et al., 2019) in which spike train correlations were seen as the input to construct simplicial complex.

With these wide applications in neuroscience, TDA may also be a promising candidate for addressing the problem regarding criticality. As a first step, we applied TDA to the firings of a network of stochastic spiking neurons in order to see how topological features would behave as the network dynamics change and if TDA can discriminate the three states of the network, i.e., sub-critical, critical, and super-critical states.

## 2 An integrate-and-fire neural network

For our purposes we considered the firing dynamics of an excitatory/inhibitory stochastic integrate-and-fire network, which has a mean-field directed percolation critical point with a fully connected structure (Girardi-Schappo et al., 2021). With this critical point, the network has three states depending on the control parameter, i.e., critical, sub-critical, and super-critical states.

Here, we briefly introduce the model. The network is composed of N neurons, and each neuron is a stochastic leaky integrate-and-fire unit with discrete time step equal to 1 ms, connected in an all-to-all graph. The membrane potential of each neuron *i*, either excitatory (E) or inhibitory (I), evolves as


(1) $$\begin{array}{c} V_{i}^{E/I}[t+1]=\\ \left[\mu_{i}V_{i}^{E/I}[t]+I_{i}^{ext}[t]+\frac{1}{N}\sum_{j=1}^{N_{E}}J_{ij}X_{j}^{E}[t]-\frac{1}{N}\sum_{j=1}^{N_{I}}W_{ij}X_{j}^{I}[t]\right]\\ \left(1-X_{i}^{E/I}[t]\right), \end{array}$$

where μ_*i*_ is the leakage parameter, $I_{i}^{ext}\left[t\right]$ is an external current, *N*_*E*_ and *N*_*I*_ are, respectively, the number of excitatory and inhibitory neurons, and the non-negative element *J*_*ij*_ (*W*_*ij*_) gives the synaptic weight between the *j*th presynaptic excitatory (inhibitory) neuron and the *i*th postsynaptic neuron. *J*_*ij*_ = 0 or *W*_*ij*_ = 0 means that the neurons are not connected. $X_{i}^{E/I}\left[t\right]$ is a stochastic Boolean variable denoting if at time t a neuron fires ($X_{i}^{E/I}\left[t\right]=1$) or not ($X_{i}^{E/I}\left[t\right]=0$). It turns to 1 with a piecewise linear sigmoidal probability Φ(*V*),


(2) $$\begin{array}{l} \Phi \left(V\right)=\left(V-\theta \right)\Gamma \Theta \left(V-\theta \right)\Theta \left(V_{S}-V\right)+\Theta \left(V-V_{S}\right), \end{array}$$

where θ is the firing threshold, Γ is the firing gain constant (Brochini et al., 2016), Θ(·) is the Heaviside function, and *V*_*S*_ = θ+1/Γ is the saturation potential. The total number of neurons in the network is *N* = *N*_*E*_+*N*_*I*_, with *p* = *N*_*E*_/*N* and *q* = *N*_*I*_/*N*.

The average over neurons of Eq. (1) yields the mean-field of the network, which presents a mean-field directed percolation (MF-DP) critical point (Girardi-Schappo et al., 2020, 2021):


(3) $$\begin{array}{l} V^{E/I}\left[t+1\right]=\left[\mu V^{E/I}\left[t\right]+I^{ext}\left[t\right]+pJ\rho_{E}\left[t\right]-qW\rho_{I}\left[t\right]\right]\left(1-\rho_{E/I}\left[t\right]\right), \end{array}$$

where *J* = 〈*J*_*ij*_〉, *W* = 〈*W*_*ij*_〉, $I^{ext}\left[t\right]=\langle I_{i}^{ext}\left[t\right]\rangle$, and μ = 〈μ_*i*_〉 are mean-field approximations, with 〈.〉 denoting average over neurons, and $\rho_{E/I}\left[t\right]=1/N_{E/I}\underset{j}{\sum }X_{j}^{E/I}\left[t\right]$. Denoting $\bar{W}=pJ-qW$ and *g* = *W*/*J* (which intuitively represents the level of inhibition), then the critical point will lie at (see Girardi-Schappo et al., 2021 for a detailed derivation)


(4) $$\begin{array}{l} {\bar{W}}_{c}=\frac{1-\mu }{\Gamma },\;g_{c}=\frac{p}{q}-\frac{1-\mu }{q\Gamma J}, \end{array}$$

where μ = 0 will be taken in this study, since this is valid, and μ>0 does not present any new phenomenology (Girardi-Schappo et al., 2021).

## 3 Methods for analysis

To analyze the firings of the network introduced above, we do topological data analysis here. First, we introduce the topological framework that is necessary for TDA.

### 3.1 Topological framework

#### 3.1.1 Simplicial complex

**Figure 1:**
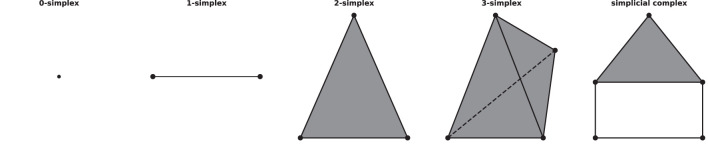
Examples of simplices and a simplicial complex, 0-, 1-, 2-, 3-simplex, and a simplicial complex (which consists of five 0-simplices, five 1-simplices, and one 2-simplex) from left to right.

A simplicial complex is a mathematical structure in algebraic topology for approximating the shape and properties of spaces, constructed from the basic geometric objects, i.e., simplices (plural of simplex). Given a set of vertices V, a n-simplex is defined as the convex hull of (n+1) affine independent vertices in V. For example, geometrically, a 0-simplex is a vertex or a point, a 1-simplex is an edge, a 2-simplex is a triangle, and so on. A simplicial complex can be constructed from simplices if they satisfy two conditions: first, if a simplex is included in the simplicial complex, then so are its faces, for example, if a triangle is a part of a simplicial complex, its three edges and vertices must be part of the complex as well; second, the intersection of any two simplices in the complex is either empty or a face of them, see Figure 1 for an example of simplices and a simplicial complex.

By defining the simplices and how they construct a simplicial complex, we can capture the structure of a specific space. In this study, we constructed the simplicial complex by treating the spike trains of neurons as the vertices in a complex, and the weight value between two vertices is equal to the dissimilarity of their corresponding spike trains. We will construct the commonly used Vietoris-Rips complex. That is, the complex is consisted of all vertices at first. Then, given a threshold, an edge between any two vertices is added to the complex if the weight value between them is less than the threshold. Moreover, a triangle or higher-dimensional simplex is added also if all its edges are in the complex. After adding all such simplices, a Vietoris-Rips complex with regard to the threshold is obtained.

#### 3.1.2 Betti numbers and persistent homology

**Figure 2:**
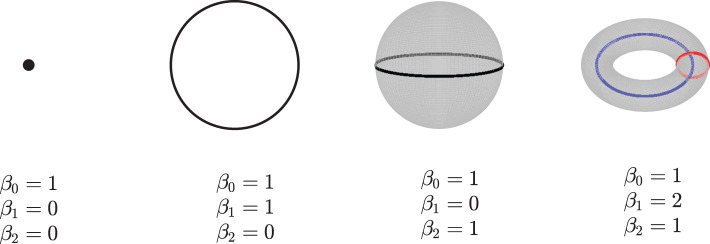
Examples of betti numbers, from left to right - a point, a circle, a 2-dimensional sphere, and a 2-dimensional torus. All of them have one connected component, so their 0-dimension betti number *β*_0_ is 1. The circle has one loop, so its 1-dimension betti number *β*_1_ is 1. Both the sphere and torus have a 2-dimension void inside; thus, their *β*_2_ are 1. Furthermore, the torus has two loops, and its *β*_1_ is equal to 2.

Given a simpilicial complex Σ, let *H*_*k*_(Σ) donate the k-dimensional homology of Σ. The dimension of *H*_*k*_(Σ) which counts the k-dimensional “holes” in Σ is called the *k*th Betti numbers, β_*k*_, i.e, β_0_ gives the number of connected components, β_1_ is the number of loops in Σ, β_2_ is the number of voids, and so on. See Figure 2 for an example. Here, we only consider the zeroth betti numbers and the first betti numbers.

**Figure 3:**
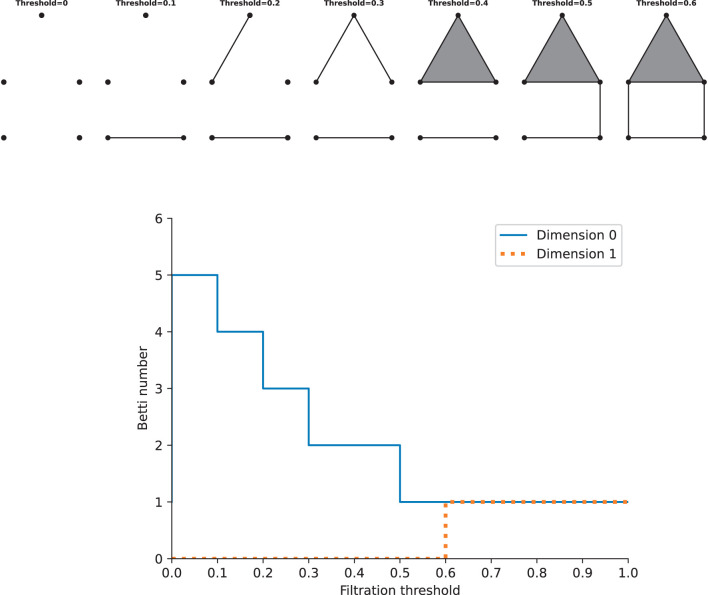
Examples of a filtration and the Betti cuvre. **Top**: the simplicial complexs with increasing filtration thresholds. **Bottom**: the Betti curves of the simplicial complexs in dimension 0 and 1. Note that this is just an illustration, the distance on the graph should not be taken as the true distance between different points.

As stated above, we can construct one complex via a single constant threshold. However, its homology could not represent the properties of the underlying space well, and it is difficult to choose a suitable threshold. Instead, we need to build a filtration, which is a nested family of complexes with regard to an increasing sequence of thresholds. At the beginning of the filtration, it consists of vertices only, i.e., there are only 0-simplices. As the threshold increases from zero to one (the maximum value of the weight), higher-dimensional simplices will appear. By this construction, we will get a nested family of complexes, and betti numbers can be computed in each complex. Calculating the homology of the complexes of all thresholds requires the persistent homology theory, which gives a way to study how the topological features such as “holes” change across the filtration. Given two parameters ϵ_0_ and ϵ_1_, if the feature at *H*_*k*_(ϵ_0_) is still present at *H*_*k*_(ϵ_1_), it is said that the feature persists from ϵ_0_ to ϵ_1_, and the features that persist more longer as the threshold increases are more significant, while others are considered as noise. The lifetime that records the “birth” (the time when holes appear) and “death” (the time when holes disappear) of features could offer more information across the filtration about the underlying data, so we can track the betti numbers in each dimension as a function of the filtration threshold, which gives rise to Betti curves. See Figure 3 for an example. The features of the Betti curves will be analyzed later.

### 3.2 Analyzing network dynamics

In the following, we applied a method based on persistent homology to analyze network dynamics using topological features of spaces built from various spike train distances (Bardin et al., 2019). Three different measures of spike train similarity are chosen here for analyzing the spiking data, i.e., the Pearson correlation, SPIKE-synchronicity (Kreuz et al., 2015), and SPIKE-distance (Kreuz et al., 2013).

**Figure 4:**
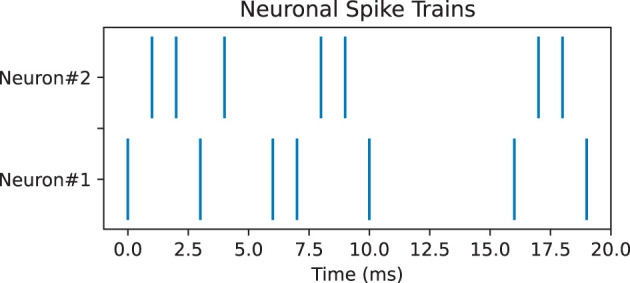
Pair of neuronal spike trains for which Pearson correlation measure is 0.17, SPIKE-synchronicity measure is 1, and SPIKE-distance measure is 0.31, with a time bin of 4 ms.

Pearson correlation can be used to measure the correlation between spike trains recorded from different neurons. It compares the spike trains of two neurons and estimates whether neurons tend to fire together or exhibit temporal dependencies, which can provide insights into the functional connectivity and information processing in neural circuits (Cohen and Kohn, 2011). However, Pearson correlation encounters many challenges such as temporal precision and time lagged correlations; thus, it is necessary to use other measures as well for complements. Here, we used two additional measures, namely, SPIKE-synchronicity and SPIKE-distance. SPIKE-synchronicity counts the simultaneous appearances of spikes in two spike trains, while SPIKE-distance measures the dissimilarity between spike trains. Notice that both Pearson correlation and SPIKE-synchronicity are bounded in [0, 1], with value 1 indicating the spike trains are identical, and they are converted to similarity measures through the function *x*↦1−*x*. In contrast, spike trains with larger SPIKE-distance value are considered more dissimilar. Importantly, these three measures all depend on the size of the time window, and for simplicity here, we used the same time-binning as in Bardin et al. (2019). For computing SPIKE-synchronicity and SPIKE-distance, we used the Python package PySpike (Mulansky and Kreuz, 2016), while Pearson correlation was computed by the Python package Elephant (Denker et al., 2018). Note that given two spike trains, these measures may have very different values, see Figure 4 for an example.

**Figure 5:**
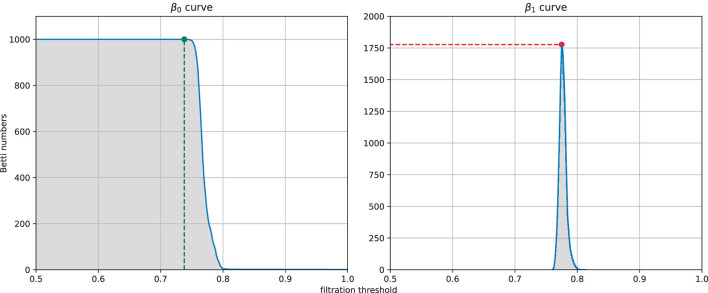
Example of the four features extracted from a filtration: the filtration threshold at which the Betti-0 curve starts to decrease (left, green), the global maximum (right, red) of the Betti-1 curve, and the area under each curve (gray area) starting from 0.

From each similarity measure, we can computed the persistent homology of the Vietoris-Rips complex of the weighted graph and then extracted four features from the zeroth and first Betti curves. The four features (see Figure 5) are the filtration value at which the Betti-0 curve starts to decrease (we called it the turning point of the Betti-0 curve), the area under the Betti-0 curve, the global maximum of the Betti-1 curve, and the area under the Betti-1 curve, which are referred to later as feature 1, 2, 3, 4, respectively. Since there are three different similarity measures, a total of 12 features can be extracted.

These features can then used as the input for machine learning in order to discern information about the global network dynamics. Following Bardin et al. (2019), machine learning here was achieved by the support vector machine (SVM, a supervised machine learning algorithm used for classification and regression) methods (Cortes and Vapnik, 1995) using a radial basis function (a real-valued function whose value depends only on the distance from some center point) kernel with *L*^2^-regularization (a kind of distance). We trained an *L*^2^-regularized SVM classifier on our selected standardized features to identify the different regimes, whose regularizing hyperparameter was selected with a tenfold cross-validation (a resampling procedure used to evaluate machine learning models on a limited data sample). During training, an 80%-20% training-testing sample partition was used, and performance of the classifier was evaluated by an accuracy score.

We considered three different classifications, aiming to see if topological features can capture some essential characteristics of each dynamical regime that help to distinct themselves from others. The first one discards the critical state and only distinguishes super-critical from sub-critical states, and the second one distinguishes all three states, while the last one distinguishes critical from the other two non-critical states. The last one is to investigate whether the critical state bears some special topological features, motivated by the variety of functional benefits of criticality in neural networks (Kinouchi and Copelli, 2006; Beggs, 2008; Shew et al., 2011; Shew and Plenz, 2013).

## 4 Results

In order to see how topological features change as the network goes through the super-critical, critical, and sub-critical states, we generated network dynamics for different values of the control parameter *g* by numerical simulations. We varied *g* from 1.20 to 1.80 in a step of 0.01 and run 10 simulations for each value of *g*. To discriminate better between critical and non-critical states later, we generated another 190 simulations for the critical point *g* = 1.50 so that there are enough samples of critical states.

**Figure 6:**
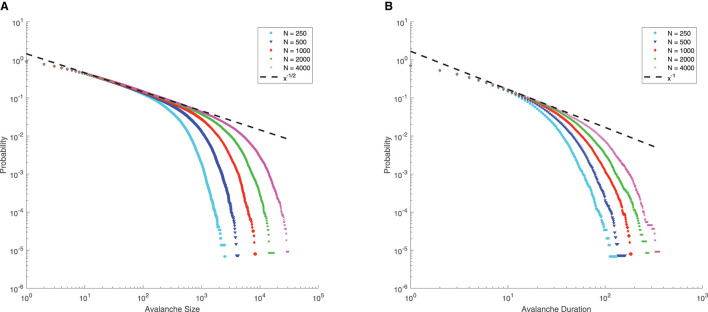
Complementary cumulative distribution function (CCDF) of neuronal avalanches at *g* = 1.5, for μ = 0, *p* = 0.8, *q* = 0.2, Γ = 10, *J* = 10. **(A)** CCDF of avalanche size; **(B)** CCDF of avalanche duration. Dashed lines indicate mean-field predictions. Both distributions start to be power-law like, followed by an exponential cutoff that grows with network size *N*, indicative of the finite-size effect. These suggest that for finite-size networks the mean-field critical point *g* = 1.5 can still be considered critical.

Note that for μ = 0, *p* = 0.8, *q* = 0.2, Γ = 10, *J* = 10, from Eq. (4), we can see that the mean-field critical point lies at *g* = 1.5, which is only reached in the limit of infinite network size. Nevertheless, for a finite-size network, we still consider *g* = 1.5 as the critical point since the distributions of avalanche size and duration at *g* = 1.5 seem to follow power-law, along with finite-size effects (the exponential decay occurs at larger size as *N* increases, see Figure 6), which is commonly considered as a signature of criticality in neuroscience (Beggs and Plenz, 2003).

For simplicity and limited computational resources, in all our numerical simulations, unless otherwise stated, we used a fixed total number *N* = 1000 neurons and set *p* = 0.8 and *q* = 0.2 as reported for cortical data (Somogyi et al., 1998). Note that though for living neural networks the ratio of excitatory to inhibitory neurons varies from 3:1 to 9:1 and remains roughly constant for different sensory areas within a species (Alreja et al., 2022), this ratio would not alter the dynamics of the network here (Girardi-Schappo et al., 2020). Each simulation was run for 1,000,000 time steps, with the first transient 10,000 steps discarded. One time step is set to be equal to a biologically plausible time, i.e., 1 ms, with the spike of a neuron takes one time step here. We set the external field *h* = 0 and used an offline driving mechanism to keep the network dynamics on, which means that a randomly chosen neuron is set to be active immediately after the activity of the network died out. See Table 1 for values of parameters used in the simulations.

**Table 1:** Default parameters used during simulations.

***N***	***p***	***q***	***J***	**Γ**	***g***	**μ**	**θ**	***I* ^*ext*^**
1000	0.8	0.2	10	0.2	1–2	0	1	1

**Figure 7:**
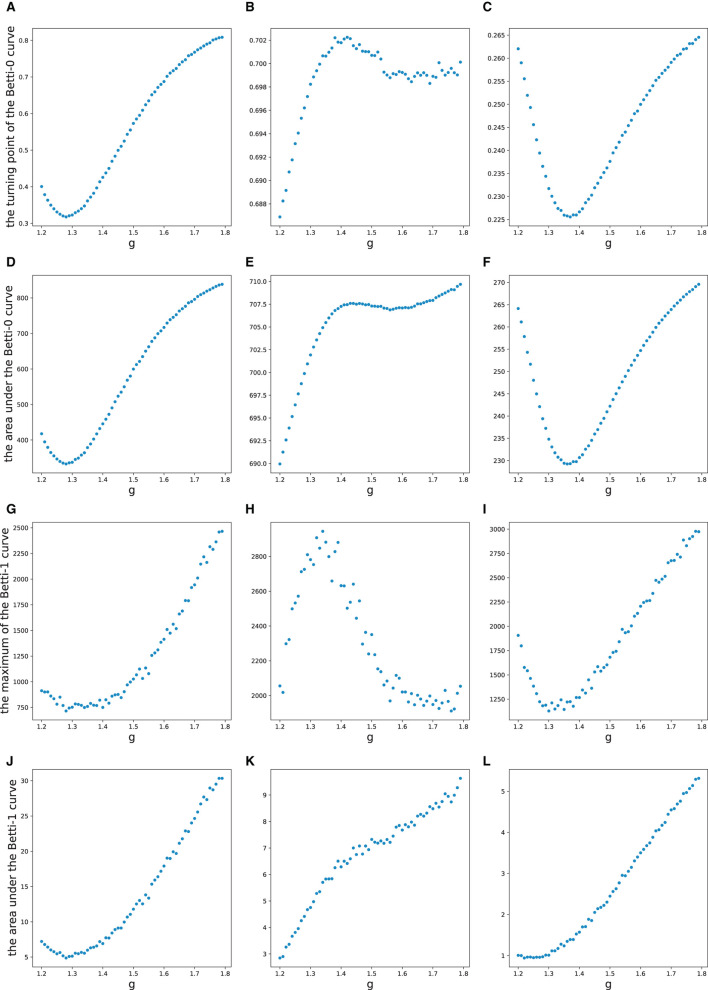
Features extracted from simulations. Values of features are averaged over 10 simulations for each value of *g*. For each row, one same feature is displayed, respectively, for three spike train similarity measures, i.e., the Pearson correlation, SPIKE-synchronicity, and SPIKE-distance. And the four different features used here are **(A–C)** the turning point of the Betti-0 curve; **(D–F)** the area under the Betti-0 curve; **(G–I)** the maximum of the Betti-1 curve; **(J–L)** the area under the Betti-1 curve.

For each simulation, we first computed three pairwise spike train similarity measures and then the persistent homology of the Vietoris-Rips complex of the weighted graph. Features discussed above from the zeroth and first Betti curves are then extracted. A total of 12 features were extracted during each simulation, see Figure 7.

From Figure 7, one can see that the four features of Pearson correlation and SPIKE-distance measure behave quite similarly. When *g*>1.5, i.e., the network is in a sub-critical state, all features increase as *g* becomes larger, while for *g* < 1.5, i.e., when the network is in a super-critical state, all features first increase and then decrease. This may have reflected that under these two dissimilarity measures, the overall distance among all neurons increases and the groups they form due to their co-activity are connected more complicatedly as inhibition gets stronger (*g* gets larger). We predict that it is easy to discriminate the super-critical, critical, and sub-critical states using features of these two measures since the curves are relatively monotonous in a certain interval around the critical state *g* = 1.5. Meanwhile, we conjecture that the smoother the curves, the easier the classification. These are verified in our classifying results, see Table 2.

**Table 2:** Mean accuracy of various classifications.

	**Pearson correlation**			**SPIKE-synchronicity**			**SPIKE-distance**		
**Features**	**Acc. 1**	**Acc. 2**	**Acc. 3**	**Acc. 1**	**Acc. 2**	**Acc. 3**	**Acc. 1**	**Acc. 2**	**Acc. 3**
1, 2, 3, 4	99.96%	98.38%	97.56%	98.52%	96.79%	91.68%	100.00%	99.42%	98.13%
2, 3, 4	99.96%	98.13%	97.25%	99.32%	97.17%	92.91%	99.83%	98.54%	97.82%
1, 3, 4	99.53%	97.92%	96.58%	98.52%	96.88%	91.55%	100.00%	99.38%	97.31%
3, 4	99.07%	96.58%	92.28%	99.07%	97.13%	93.20%	99.11%	96.29%	93.23%
1, 2, 4	100.00%	98.75%	98.10%	95.93%	93.71%	81.20%	100.00%	99.96%	98.45%
2, 4	99.96%	98.42%	98.07%	93.86%	92.38%	80.73%	99.96%	99.08%	98.29%
1, 4	99.75%	98.58%	97.47%	94.49%	92.54%	80.44%	100.00%	99.71%	97.47%
4	97.84%	95.33%	88.99%	89.28%	87.46%	73.23%	98.14%	95.21%	91.42%
1, 2, 3	99.96%	98.67%	97.41%	95.38%	91.75%	78.70%	99.32%	98.38%	97.91%
2, 3	100.00%	98.46%	97.31%	94.45%	92.42%	76.36%	98.43%	96.67%	96.99%
1, 3	99.58%	97.92%	97.03%	94.49%	91.54%	76.14%	99.15%	98.46%	96.52%
3	93.52%	91.38%	77.31%	87.08%	85.38%	73.16%	92.37%	89.54%	79.78%
1, 2	100.00%	98.96%	98.83%	93.39%	89.88%	76.93%	100.00%	99.79%	98.99%
2	100.00%	99.08%	98.83%	80.00%	79.96%	78.01%	85.13%	82.25%	97.94%
1	100.00%	98.79%	98.32%	77.33%	74.13%	70.73%	85.08%	83.08%	97.34%

Features 1–4 refer, respectively, to the turning point of the Betti-0 curve, the area under the Betti-0 curve, the maximum of the Betti-1 curve, and the area under the Betti-1 curve. Three different similarity measures (Pearson correlation, SPIKE-synchronicity, and SPIKE-distance) are separately considered here, with each having 15 combinations of the four features used for classification. Acc. 1, Acc. 2, and Acc. 3 refer to the mean accuracy averaged over 20 simulations of three different classifications, i.e., distinguishing super-critical from sub-critical states, distinguishing all three states, and distinguishing critical from non-critical states, respectively.

However, for SPIKE-synchronicity measure, things are quite different. Overall, as *g* increases, feature 1 first increases and then decreases up to a certain level, feature 2 behaves similarly but it increases again not far from the critical point *g* = 1.5, feature 3 first increases and then seems to decrease back to the starting level, while feature 4 increases all the time. We have no idea why it behaves so differently from the other two measures. A possible explanation is that SPIKE-synchronicity measure reflects more accurately the synchronous activity of neurons and it may have been influenced a lot by the special role of criticality. This requires more future study that provides deep insights into the meanings of these features. Nevertheless, we conjecture that compared to the other two measures, the classification accuracy would drop a bit when using the four features of SPIKE-synchronicity measure, which again can be confirmed in Table 2.

Of course, different combinations of features would result in different accuracy of classification. But overall, we find that a very high accuracy of classification would be gained when using features whose changing curve over *g* is smooth and relatively monotonous in a certain interval around the critical point *g* = 1.5, as can be seen from Figure 7 and Table 2.

## 5 Discussion

In this study, we applied TDA to a stochastic excitatory/inhibitory network model that has a mean-field critical point (Girardi-Schappo et al., 2020, 2021). With the existence of a critical point, the network can be super-critical, critical, or sub-critical. We are interested in the behaviors of topological features of the network as it changes among these different dynamical regimes. Three different similarity measures are considered here for constructing simplices that are basic elements of TDA, namely, Pearson correlation, SPIKE-synchronicity, and SPIKE-distance. Four features regarding Betti curves are calculated for analysis, which are then used as inputs for machine learning to discriminate the three states of the network. However, it should be noted that the behaviors of such features are yet hard to explain. Betti-0 number counts the number of connected components in the complex, and the turning point of and the area under Betti-0 curve reflects, respectively, the smallest distance and the overall distance of all vertices. Betti-l number counts the number of 1-dimensional “holes” in the complex. The global maximum of and the area under the Betti-1 curve are difficult to understand, and they may have reflected the underlying complicated distributions of local groups due to their firing activity. Why these features vary as in Figure 7 remains to be uncovered.

Nevertheless, our study shows that topological features can reflect many important implicit aspects of network dynamics, and some of these features make it possible to classify different dynamical regimes of the network easily. This is a promising direction for developing new methods for assessing the criticality of neural systems. Nevertheless, the study here bares many limitations. First of all, we only considered a special network model in its mean-field case, and more realistic ones with various connections could be studied. Second, there is only one control parameter *g* in our network model, which reduces the difficulty of classification sufficiently, as compared to the work of Bardin et al. (2019), where there are two control parameters. In fact, for our case, there are simpler classification methods. For example, using the mean network activity and its variance as the input for machine learning can classify the three regimes with high accuracy as well, see Table 3. Then why do we choose TDA? Mainly because we think TDA is a method that is worth developing since it contains more underlying information, and it uses only the firing data which is not limited to the model used here. When more complicated models and more regimes are considered, classification is very likely to become much more difficult, and TDA could be more useful then. Of course, this requires more future studies.

**Table 3:** Mean accuracy of classification using the mean network activity and its variance.

**Features**	**Acc. 1**	**Acc. 2**	**Acc. 3**
1, 2	100.00%	99.42%	99.68%
2	99.41%	96.71%	91.36%
1	100.00%	100.00%	100.00%

Features 1 and 2 refer to the mean network activity $\bar{\rho }$ and its variance, respectively. For each sample, there are 990,000 time steps, $\bar{\rho }$ is calculated as the firing rate $\rho \left(t\right)=\underset{i}{\sum }X_{i}\left[t\right]$ averaged over all time steps. On the other hand, every 10,000 time steps a mean firing rate is calculated and then the variance of these mean firing rates are used for approximating the variance of $\bar{\rho }$. Acc. 1, Acc. 2 and Acc. 3 are the same as in Table 2.

Apart from that, the resolution of the control parameter *g* in this study is chosen to be 0.01. Though we think it is relatively small, it may happen that as the resolution gets higher and higher, the classification would ultimately fail due to the lack of enough samples. Nevertheless, up to a suitable resolution, the classification should work, and its accuracy would be still high as long as there are enough samples.

Additionally, we did not provide any control for the results. For example, we did not show how TDA measures behave on shuffled data. As we checked, after shuffling the firing data, the correlations between neurons reduced greatly, and consequently, the elements of distance matrices became very close to each other, which resulted in an extremely long time of the computation of the features that is beyond our computational resources. But based on these distance matrices, we think the features of such shuffled data would not display much changes as compared to the original data.

Moreover, we applied three commonly used similarity measures for analyzing spike trains, but there is no guarantee that these measures capture all or the most important aspects of network dynamics best, so are the four features we chose for analysis. More measures and features could be explored to reflect network dynamics better. More future work could be devoted to investigating whether there are topological features (or combinations of them) that reflect the super-critical, critical, and sub-critical states of neural networks unambiguously, which is very meaningful given the various functional benefits (Kinouchi and Copelli, 2006; Beggs, 2008; Shew et al., 2011; Shew and Plenz, 2013) that criticality can offer to neural systems.

## Data availability statement

The raw data supporting the conclusions of this article will be made available by the authors, without undue reservation.

## Author contributions

XB: Conceptualization, Data curation, Formal analysis, Software, Writing—original draft, Writing—review & editing. CY: Conceptualization, Methodology, Software, Validation, Visualization, Writing—review & editing. JZ: Funding acquisition, Project administration, Writing—review & editing.
